# Drug-induced Angle-Closure Glaucoma

**DOI:** 10.5005/jp-journals-10008-1100

**Published:** 2012-10-16

**Authors:** Aruj K Khurana, Bhawna Khurana, Ashok K Khurana

**Affiliations:** 1 Senior Professor, Post Graduate Institute of Medical Sciences, Rohtak, Haryana, India; 2 Post Graduate Institute of Medical Sciences, Rohtak, Haryana, India; 3 Post Graduate Institute of Medical Sciences, Rohtak, Haryana, India

**Keywords:** Acute angle closure, Drug-induced glaucoma.

## Abstract

Drug-induced angle-closure glaucoma is an important entity for the ophthalmologist as well as the general physician as it represents a preventable cause of potential blindness. This brief review highlights the fact that a high index of suspicion, in a susceptible individual followed by confirmation on appropriate imaging modality (UBM, ultrasound or anterior segment OCT) can alleviate the threat to sight and also help to institute appropriate therapy.

## INTRODUCTION

Drug-induced angle-closure glaucoma is an important entity for the ophthalmologist as well as the general physician as it represents a preventable cause of potential blindness.^1^ Acute angle-closure glaucoma can develop in a susceptible individual by various classes of drugs.^2^ Practitioners using any of these drugs should be aware of their potential to cause acute angle closure, such that a patient presenting with signs or symptoms of acute angle closure should be immediately referred to an ophthalmologist.^3^ Drugs implicated in the causation of acute angle-closure glaucoma (AACG) include sulfa-based drugs, adrenergic drugs and drugs with anticholinergic effects. It is important to understand that the mechanism of causation of AACG is different for each of these drugs because the management strategy differs as per the pathophysiology. Ultrasound biomicroscopy helps in clinching the diagnosis by ruling out other causes of shallow anterior chamber, such as accommodative spasm and primary angle closure.^4^

### Mechanism of Drug-induced AACG

*Sulfa drugs* like topiramate, a sulfamate substituted mono-saccharide antiepileptic agent precipitates AACG by way of inducing ciliary body edema. The underlying mechanism has been well characterized by ultrsound technology. Drug-induced changes in membrane potential have been hypothesized to cause ciliary body edema,^7^ leading to relaxation of zonules and resultant lens thickening. Anterolateral rotation of the ciliary body about its attachment to the scleral spur leads to anterior displacement of the lens and iris and concomitant shallowing of anterior chamber. Concomitant choroidal detachment and supraciliary effusion are known to occur. The fact that effusion occurs only in a few patients taking topiramate and, more importantly, it typically occurs on doses well within the normal therapeutic range and in patients with normal anterior chamber depth suggests an idiosyncratic etiology.^8^ No known risk factors exist for this syndrome.^9^ Other sulfa-based drugs known to be associated with AACG: Acetazolamide, hydrochlorothiazide and cotrimoxazole.^10^

*Anticholinergic drugs* implicated in the causation of AACG include atropine, homatropine, cyclopentolate and ipratropium bromide.^10,11^ Atropine is often used to treat bradycardia, especially related to general anesthesia. Postoperative AACG has been reported in patients after general anesthesia for abdominal, orthopedic, facial and endoscopic surgery.^12^

*Botulinum toxin* has been reported as a cause of AACG. The mechanism was postulated as diffusion toward the ciliary ganglion and impedance of cholinergic innervation of the pupil, following its injection around eyelid for blepharospasm.^13^

*Cholinergic agents,* such as pilocarpine, acetylcholine and carbachol, can induce AACG by causing forward movement of iris lens diaphragm,^10^ especially in eyes with zonular weakness and exfoliation syndrome.^14^

*Adrenergic agents* can precipitate an attack of AACG through ocular^15^ as well as systemic route of administration (surgical anesthesia, anaphylactic shock, ventricular fibrillation, nasal administration for epistaxis and bronchodilators). Ocular agents cause mydriasis which can precipitate an attack of acute AACG in predisposed individuals with shallow anterior chambers.^5^ Stimulation of ciliary body beta-2 receptors increases aqueous secretion. Some drugs have an indirect sympathomimetic activity that can induce AACG, such as amphetamines, antidepressants and cocaine.^6^

*Antidepressants,* such as tri and tetracyclic depressants and SSRIs, have been associated with AACG in susceptible individuals due to their cholinergic action.^16^ Supraciliary effusion seen on ultrasonography has been identified as the pathogenetic mechanism.^17^

*Anticoagulant therapy,* in the form of heparin as well as low molecular weight heparin (enoxaparin, warfarin), can cause AACG by inducing massive vitreous, choroidal or subretinal hemorrhage. Risk factors for the same include anticoagulants, exudative age related macular degeneration and nanophthalmos.^18^

*Histamine H1 and H2 receptor antagonists,* such as chlorpheniramine and cimetidine respectively, have a weak anticholinergic effect, which can induce mydriasis and AACG in predisposed patients.^19^

*Investigative modalities for diagnosis of drug-induced AACG include* high frequency ultrasound biomicroscopy, anterior segment ocular coherence tomography and B-scan ultrasound. They help to establish and document the underlying mechanism^20^ and prognostication of drug-induced AACG. Patients with severe ciliochoroidal effusion on ultrasound B scan have been noted to have the severe visual loss. highest myopic reading and maximum increase in intraocular pressure.^21^ Fluorescein angiography further helps to confirm the diagnosis by demonstrating transient lobular choriocapillaris hypoperfusion related to choroidal thickening.^22^

*Management of drug-induced AACG* depends on the underlying pathogenic mechanism and the presence or absence of pupillary block. which helps us to decide whether peripheral iridectomy would be curative or not.

Management of topiramate related AACG requires stopping the drug in concert with the prescribing physician. because decreasing the dose by as little as 50 mg may exacerbate preexisting systemic conditions.^23^ In all reported cases, none has subsided without discontinuation of the drug. It is important to realize that most glaucoma cases resolve without miotics or iridotomy because pupillary block is not the cause of angle closure.^21^ Topical cycloplegic agents probably lower intraocular pressure by retracting the ciliary processes, along with topical-blockers and oral pressure-lowering agents. If unrecognized as a drug-related event, serious outcomes can occur (seven cases of permanent visual loss have been reported).^3^ For severe cases associated with very high intraocular pressures, the combination of mannitol and methylprednisolone can induce a rapid improvement which suggests the inflammation may be a part of the pathogenesis of topiramate induced AACG.^24^

Similarly in case of anticoagulants, the drug needs to be discontinued along with AACG management. Surgery may be required to drain choroidal effusion or hemorrhage.^25^

*Prevention of drug-induced AACG,* unfortunately does not have a straight forward solution because patients who are prescribed these drugs by physicians are not routinely sent to an ophthalmologist for a prior complete eye examination including gonioscopy to rule out preexisting narrow angles. Referring each patient for a prior ophthalmic evaluation may not be practically feasible. However, with increasing recognition of this entity, it is important for the physician to be on the look out for a predisposed patient by way of clues, such as patients wearing thick glasses that magnify objects suggesting a hypermetropic error. Ophthalmological consultation is warranted in a predisposed patient before starting treatment with drugs capable of potentiating AACG.^26^ A quick lateral torch light examination can also be carried out to rule out narrow angles. Ates et al recommend practising an oblique penlight illumination test by anesthesiologists to estimate anterior chamber depth and determine the population at risk before administering anticholinergic anesthetic drugs.^27^ Patients at risk for AACG in the postoperative period can be administered topical pilocarpine therapy to prevent any attack. Since symptoms of AACG may be overlooked or misinterpreted in a sedated or comatose patient, any patient who has a red eye and a subjective vision loss postoperatively should be examined urgently.^3^

Most importantly, if a patient on any of the implicated drugs reports back to the physician with symptoms suggestive of AACG, such as acute painful red eye or blurred vision, an immediate ophthalmological referral is warranted.

## CONCLUSION

Drug-induced acute angle-closure glaucoma is a potentially avoidable cause of vision loss which requires vigilance on part of the physician and the ophthalmologist. A high index of suspicion, in a susceptible individual followed by confirmation on appropriate imaging modality (UBM, ultrasound or anterior segment OCT) can alleviate the threat to sight and also help to institute appropriate therapy.
